# A Novel Mechanism of STAT3 Activation by Oncogenic Signaling

**DOI:** 10.3390/cells15090755

**Published:** 2026-04-23

**Authors:** Magesh Muthu, Jaganathan Venkatesh, Kaladhar B. Reddy, Arun K. Rishi

**Affiliations:** 1 John D. Dingell V.A. Medical Center, Detroit, MI 48201, USA; fg6627@wayne.edu (M.M.); jaganvibt@gmail.com (J.V.); 2 Department of Oncology, Karmanos Cancer Institute, Wayne State University, Detroit, MI 48201, USA; 3 Department of Pathology, Karmanos Cancer Institute, Wayne State University, Detroit, MI 48201, USA; kreddy@med.wayne.edu

**Keywords:** CARP-1/CCAR1, IL-6, STAT3, p21Rac1, EGF

## Abstract

**Highlights:**

**What are the main findings?**

- CARP-1 interacts with STAT3 and small GTPase p21Rac1.
- STAT3 (441-480) regulates IL-6 or EGF-induced STAT3 activation and nuclear translocation.

**What are the implications of the main finding?**

- CARP-1/STAT3/p21Rac1 complex is a transducer of pro-inflammatory signaling.
- STAT3 (456-465) is a novel target for STAT3 inhibition.

**Abstract:**

CARP-1, a perinuclear phospho-protein, is a biphasic regulator of cell survival and apoptosis signaling. We previously found that UV cross-linking of proteins from HeLa cervical cancer cells resulted in STAT3 interacting with the CARP-1 (614–638) peptide. Mutagenesis and co-IP-WB experiments revealed that CARP-1 interacts with a 40-amino-acid epitope from positions 441–480 (CE Epitope) located in the STAT3 DNA-binding domain. Overexpression of mutant STAT3 with in-frame deletion of the CE epitope (Gst-STAT3 (ΔCE) mutant), but not Gst-STAT3 (WT), failed to translocate to the nucleus in IL-6-treated cells. The small GTPase p21Rac1 interacts with and regulates STAT3 activation and nuclear translocation. Here we report the interaction of p21Rac1 with the CE epitope of STAT3 and the CARP-1 (600–650) region, suggesting that CARP-1 is part of a dynamic STAT3-p21Rac1 complex that functions in STAT3 activation and nuclear translocation. Expression of a STAT3 (ΔCE) mutant abolished STAT3 Y705 phosphorylation in cells that were treated with EGF or IL-6. Fine mapping revealed that scrambling the CE epitope peptide or a small peptide from positions 456–465 within the CE epitope resulted in abrogation of STAT3 Y705 phosphorylation by IL-6. Moreover, STAT3 phosphorylation by EGF or IL-6 was diminished in multiple CARP-1 null cancer cells. Importantly, incubation of a TAT-tagged STAT3 (454–467) peptide but not its scrambled version resulted in a reduction in STAT3 Y705 phosphorylation by IL-6/EGF. Taken together, our data demonstrates that the STAT3 CE epitope interacts with CARP-1 and p21Rac1, harbors novel sequences that activate STAT3 and promotes its nuclear translocation by IL-6/EGF.

## 1. Introduction

Signal transducer and activator of transcription (STAT)3 is a member of the STAT family of transcription factors, which are expressed in almost all vertebrate cells. STAT3 regulates basic cell functions, including cell growth, survival, differentiation, regeneration, immune response, and respiration [1]. STAT3 is transiently activated in normal tissues. STAT3 is aberrantly activated/induced by oncogenic cytokines such as IL-6, as well as EGFRs, Src, and Janus kinases (JAKs). Activation of STAT3 involves phosphorylation at tyrosine (Y) 705 by JAKs or cSrc. STAT3 Serine (S) 727 phosphorylation can also influence its activity. Activated STAT3 dimerizes, localizes to the nucleus, and regulates transcription of its target genes. As aberrant STAT3 activation is associated with cancer formation, progression, and metastasis, STAT3 pathways are considered promising targets for cancer therapy [1,2].

CARP-1/CCAR1 is a peri-nuclear phospho-protein that is a biphasic regulator of cell growth, survival, and apoptosis signaling [3,4]. CARP-1 functions as a co-activator of growth signaling by steroid thyroid nuclear receptors and oncogenic signaling by β-catenin. It co-activates the glucocorticoid receptor for adipocyte differentiation, the anaphase-promoting complex cyclosome (APC/C) E3 ligase, tumor suppressor p53 for cell cycle control and apoptosis, and a splicing factor of Fanconi anemia FANCA protein for DNA repair [4,5,6,7,8]. CARP-1 is an oncogene in liver cancers, where it enhances growth and metastasis and promotes resistance to anti-PD1 therapy [9]. CARP-1 directly binds the NF-κB-activating kinase IκB kinase subunit γ (IKKγ or NF-κB essential modulator, aka NEMO) and regulates the chemotherapy-activated canonical NF-κB pathway [10]. Our recent LC/MS/MS and co-IP-WB studies revealed that CARP-1 (600–650) interacted with STAT3 [11]. Here, we mapped the STAT3 epitope that interacts with CARP-1 (referred to as the CE epitope) and tested the hypothesis that the CE epitope is required for STAT3 activation by diverse upstream signals.

## 2. Materials and Methods

Materials: The cell culture media, DMEM, RPMI-1640, and antibiotics (streptomycin and penicillin) were purchased from Invitrogen Co. (Carlsbad, CA, USA). Ham’s F-12 medium was purchased from Life Technologies, Inc., Grand Island, NY, USA, and fetal bovine serum (FBS) was obtained from Denville Scientific Inc. (Metuchen, NJ, USA) and Peak Serum Inc. (Atlanta, GA, USA). Purified IL-6, soluble IL-6 receptor α, and EGF were obtained from Peprotec Inc., Cranbury, NJ, USA. The plasmids pDONOR/STAT3 (Human) and pEGFP-human STAT3 were obtained from Addgene (Cambridge, MA, USA). Clinical-grade Adriamycin was obtained from the pharmacy, Karmanos Cancer Institute, Wayne State University, Detroit, MI, USA. Dimethyl sulfoxide (DMSO), 2-deoxy-glucose, 3-4, 5-dimethyltiazol-2-yl-2.5-diphenyl-tetrazolium bromide (MTT), and anti-actin antibodies were purchased from Sigma Chemical Co., St. Louis, MO, USA. The affinity-purified, anti-CARP-1 (α1 and α2) polyclonal antibodies have been described [3]. Antibodies for Gst-tag, Flag-tag, Myc-tag, STAT3, Y705 phospho-STAT3, STAT1, Y701 phospho-STAT1, p21Rac1, βActin, and αtubulin were purchased from Cell Signaling, Beverley, MA, USA. Anti-Lamin and GAPDH antibodies were purchased from Santa Cruz Biotech, Santa Cruz, CA, USA.

### 2.1. Cloning of cDNAs

The plasmid for the expression of myc-His-tagged wild-type CARP-1 (clone 6.1.2) and myc—His-Tagged CARP-1 (Δ600–650) has been described before [3,11]. Additional pcDNA-based plasmids for the expression of Gst, Gst-STAT3 (WT), Gst-STAT3 (Δ441–480), Gst-STAT3 (Δ441–465), Gst-STAT3 (Δ456–480), Gst-STAT3 (1–200), Gst-STAT3 (181–400), Gst-STAT3 (381–600), Gst-STAT3 (581–780), Gst-STAT3 (411–450), Gst-STAT3 (441–480), Gst-STAT3 (471–510), Gst-STAT3 (501–540), Gst-STAT3 (531–570), Gst-STAT3 (561–600), Gst-STAT3 (EVYHQ > QLCQP), Gst-STAT3 (EVYHQ > QLCQP), Gst-STAT3 (ICQMPNA > VSQLPSG), Gst-STAT3 (441–480 Scrambled), Gst-STAT3 (EVYHQ > QLCQP; 456–465 Scrambled; ICQMPNA > VSQLPSG), Gst-STAT3 (456–465 Scrambled), Gst-STAT3 (VVVIS > AAADL), Gst-STAT3 (THSLP > PSLDT), and Gst-STAT3 (H457L) were generated by standard molecular biological and cloning manipulations. All the recombinant plasmids were sequenced to confirm the accuracy and validity of various inserts/epitopes.

### 2.2. Cell Lines and Cell Culture

Routine maintenance and culture of MDA-MB-468, MDA-MB-231 (lacking ER and with mutant p53) human triple-negative breast cancer (TNBC) cells, 4T1 murine TNBC cells, human cervical cancer HeLa cells, human colon cancer HCT-116 cells, and human embryonic kidney (HEK) 293T cells were carried out as described previously [3,10,11]. The generation and characterization of CARP-1-/- (CARP-1 KO) HeLa cells have been described previously [10]. CARP-1 KO human colon cancer HCT-116 and mouse TNBC 4T1 cells were generated by Ubigene Inc., Guangzhou, P.R.China, on a fee-for-service basis using CRISPR technology. STAT3-/- Human Colon Cancer A4 cells were obtained from Dr. George Stark (Cleveland Clinic, Cleveland, OH, USA) and have been described before [12,13]. The stable sublines were generated by transfecting the MDA-MB-468, HEK293T, and A4 (STAT3-/-) cells with the above-described various recombinant plasmids expressing Gst-STAT3 fusion proteins, followed by selection in the presence of 800 μg/mL neomycin using the described methods [3]. The cell lysates from wild-type, untransfected cells, neomycin-resistant pools, or individual sublines were then subjected to IP and WB analyses, as described below.

### 2.3. Immunoprecipitation (IP), Gst-Pulldown, and Western Blot (WB) Assays

Logarithmically growing cells were either untreated or treated with different agents for various time periods. The cells were transfected with different plasmids, and in some cases, stable sublines were selected in the presence of neomycin, as previously described [11]. For immunoprecipitation, cells were lysed in RIPA (1×) buffer to prepare protein extracts, and IP was performed by incubating approximately 1 mg of the protein lysate with appropriate antibodies. The immuno-complexes were pelleted by centrifugation at 800× *g* for 2 min, and the pellet was washed two–four times with 100–200 microliters of RIPA buffer with 0.5 M NaCl. In some cases, additional washes with RIPA buffer containing 1 M NaCl were performed. After the final wash, the antibody-bound protein complexes were spun and then re-suspended in SDS loading buffer for electrophoresis on 12–15% SDS PAGE, followed by WB with appropriate antibodies.

### 2.4. Synthesis of Peptides

In total, 40 mg of ≥95% pure STAT3 (454–467) wild-type and scrambled peptides were commercially synthesized by ABI Scientific, Sterling, VA, USA. Each peptide had an N-terminal 6xHis and TAT epitope and a C-terminal Flag epitope. The peptide sequences are as follows:

STAT3 (454–467) Scrambled: MHHHHHHYGRKKRRQRRRVLNILSHSIVETPVDYKDDDDK

STAT3 (454–467): MHHHHHHYGRKKRRQRRRLETHSLPVVVISNIDYKDDDDK

Each peptide was dissolved in sterile water, and cells were incubated with 300–500 micrograms per ml of the peptide solution for various time periods, followed by immunofluorescent staining of cells, as described below, or by cell lysis for WB analysis of activated and total STAT3, STAT1, and actin proteins, as detailed above.

### 2.5. Immunofluorescence Staining and Confocal Microscopy

Immunofluorescence and microscopy analyses were performed as described previously [10,14]. Briefly, cells were plated onto chamber slides 1 day before treatment. Following treatment of cells with the relevant agents (peptides or IL-6), the adherent cells were fixed with 5% formaldehyde for 10 min and then washed with PBS, followed by blocking (0.5% NP-40, 5% milk powder, 1% fetal bovine serum) for 30 min. After a single wash with PBS, cells were incubated with primary antibodies for 45 to 60 min, washed with PBS, and then incubated with secondary antibodies for another 45 to 60 min. They were then washed with PBS and mounted with 0.1 μg/mL DAPI (4′,6′-diamidino-2-phenylindole)-containing mounting solutions. For confocal imaging, cells were first fixed with PFA, stained with GST-tag antibodies (green) for STAT3, Flag-tag antibodies (green) for STAT3 peptides, and DAPI (blue) for nuclear staining and then imaged by confocal microscopy.

### 2.6. Statistical Analyses

The statistical analyses were performed using Prism 6.0 software as needed. The data were expressed as mean ± SEM and analyzed using a two-tailed Student’s *t*-test or one-way ANOVA followed by a post hoc test. *p*-values of <0.05 were considered statistically significant.

## 3. Results

**Figure 1:**
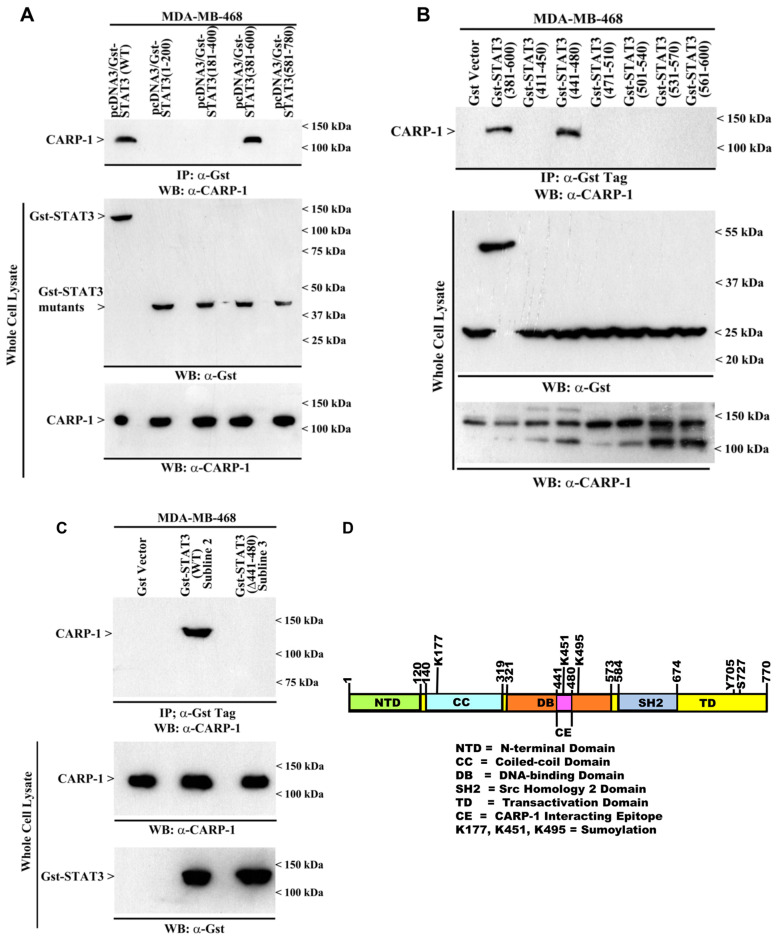
CARP-1 interacts with STAT3 (441–480). (**A**–**C**). Protein complexes from the indicated cells expressing noted Gst-STAT3 fusion proteins were immunoprecipitated (I.P.) with anti-Gst tag antibodies, followed by the analysis of the immunocomplexes by Western blot (W.B.) using anti-CARP-1 antibodies (upper blots). The membranes containing proteins from whole-cell lysates were then probed with anti-CARP-1 or anti-GST antibodies to detect the respective proteins. Arrowheads on the left or right side of each blot indicate the presence of the proteins and molecular weight markers, respectively, in panels (**A**–**C**). (**D**) Schematic illustration of STAT3 protein showing its N-terminal, coiled-coil, DNA-binding, Src-homology 2 (SH2), and C-terminal transactivation domain. The STAT3 epitope that interacts with CARP-1 is denoted as CE.

STAT3 (441–480) interacts with CARP-1. We previously found that CARP-1 (600–650) interacted with STAT3, and that treatment with genotoxic chemotherapy, Adriamycin, or expression of CARP-1 (S^626^, T^627^/AA) interfered with CARP-1 interaction with STAT3 [11]. Here, we further determined the minimal epitope of STAT3 that interacts with CARP-1. Our co-IP-WB analysis of protein lysates from TNBC MDA-MB-468 cells expressing different Gst-STAT3 mutants revealed that CARP-1 interacts with the STAT3 (381–600) region (Figure 1A). Further co-IP-WB analyses of the protein lysates derived from cells expressing overlapping, Gst-tagged 40 amino acid peptides derived from the STAT3 (381–600) region revealed a CARP-1 interaction with the STAT3 (441–480) peptide, while STAT3, but not the STAT3 mutant protein with in-frame deletion of amino acids 441–480 (STAT3 Δ441–480), interacts with CARP-1 (Figure 1B,C). The STAT3 amino acids 441–480 that interact with CARP-1 are denoted as the CE epitope and are part of the DNA-binding domain of STAT3 (Figure 1D).

**Figure 2:**
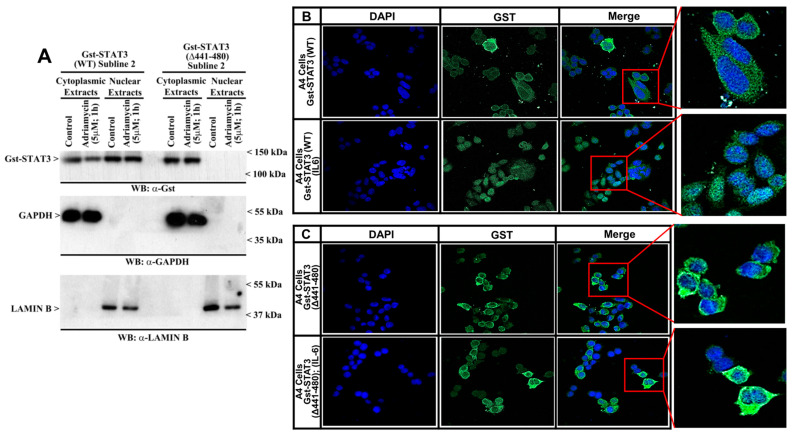
STAT3 (Δ441–480) does not translocate to the nucleus. (**A**) The TNBC MDA-MB-468 cells expressing noted Gst-STAT3 plasmids were either treated with DMSO (Control) or treated with the indicated agent at the noted time and dose. Cellular proteins were first separated into cytosolic and nuclear fractions. The cell lysates were then analyzed by WB for levels of Gst-STAT3, Lamin B, and GAPDH proteins, as described in the Methods. Arrowheads on the left or right sides of each blot indicate the presence of proteins and molecular weight markers. (**B**,**C**) A4 colon cancer cells stably expressing Gst-STAT3 (wild-type) or Gst-STAT3 (Δ441–480) protein were either untreated or treated with a combination of IL-6 (50 ng/mL) and sIL6R (10 ng/mL) for 30 min. Cells were then processed for immunofluorescence staining for Gst (green) and DAPI (blue), as detailed in the Methods. Images were taken using a Zeiss LSM 510 Meta NLO (Oberkochen, Germany), Magnification: 63×. Images in the square boxes on the right of the (**B**,**C**) panels are shown at 250× magnification.

STAT3 (Δ441–480) does not translocate to the nucleus: MDA-MB-468 TNBC or A4 STAT3 KO colon cancer cells were first transfected with Gst-STAT3 (WT), GST-STAT3 (Δ441–480), Gst-STAT3 (Δ441–465), and Gst-STAT3 (Δ456–480) plasmids, followed by the selection of multiple, neomycin-resistant sublines, as described in the Methods (Supplementary Figure S1). Next, MDA-MB-468 TNBC sublines expressing Gst-STAT3 (WT) or Gst-STAT3 (Δ441–480) were either untreated or treated with Adriamycin. We previously noted that Adriamycin treatment caused a decrease in the interaction of CARP-1 and STAT3 [11]. To determine whether and to what extent the presence of Adriamycin will also alter STAT3 translocation, we treated cells with Adriamycin prior to isolating the cytoplasmic and nuclear protein extracts. Cellular proteins were separated into cytosolic and nuclear fractions. These nuclear and cytosolic protein extracts were then analyzed by WB for the presence of Gst-STAT3, GAPDH, and Lamin B proteins. As shown in Figure 2A, Gst-STAT3 (WT) was present in both the cytoplasmic and nuclear extracts, while Gst-STAT3 (Δ441–480) was noted only in cytoplasmic extracts. The data in Figure 2A suggest that while Adriamycin’s presence may not interfere with STAT3 translocation, deletion of the CE epitope interferes with nuclear translocation of STAT3. An absence of STAT3 (Δ441–480) translocation to the nucleus was further validated by immunofluorescence analyses. For this purpose, we used A4 colon cancer cells expressing Gst-STAT3 (WT) or Gst-STAT3 (Δ441–480), which were either untreated or treated with cytokine IL-6. The cells were fixed, and the presence of Gst-STAT3 was monitored by immunofluorescence using anti-Gst antibodies, as described in the Methods. Consistent with the absence of nuclear translocation of the Gst-STAT3 (Δ441–480) in Figure 2A, immunofluorescence data also revealed localization of Gst-STAT3 (Δ441–480) in the cytoplasm, while Gst-STAT3 (WT) was present in both the nuclear and cytoplasmic compartments of cells that were either untreated or treated with IL-6 (Figure 2B).

**Figure 3:**
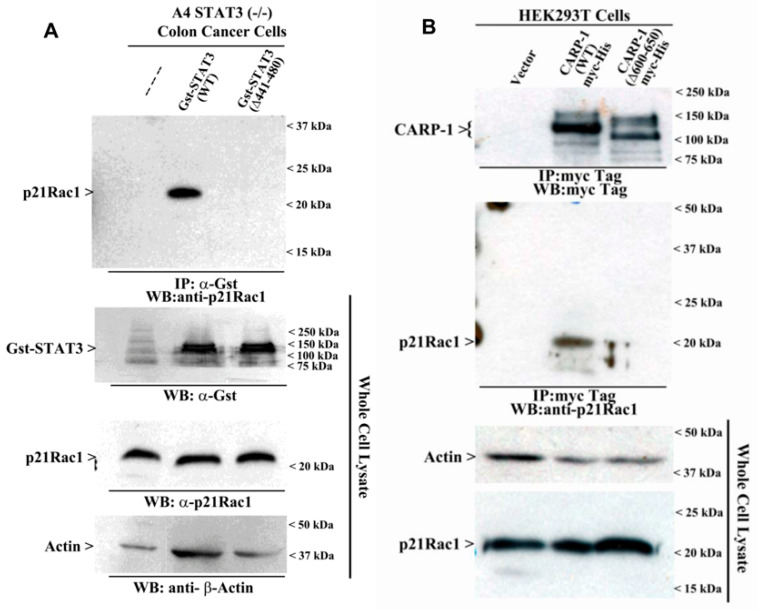
Small GTPase p21Rac1 Interacts with STAT3 (441–480) and CARP-1 (600–650). (**A**,**B**). Protein complexes from the indicated cells expressing noted Gst-STAT3 or CARP-1 myc-His proteins were immunoprecipitated (I.P.) with anti-Gst tag (**A**) or anti-myc tag (**B**) antibodies, followed by the analysis of the immunocomplexes by Western blot (W.B.) using anti-p21Rac1 (Upper and middle blots in (**A**) and (**B**), respectively) or myc-tag antibodies (Upper blot in (**B**)). The membranes containing proteins from whole-cell lysates were then probed with anti-Gst antibodies (**A**) or anti-p21Rac1 and anti-Actin antibodies (**A**,**B**) to detect the respective proteins. Arrowheads on the left or right sides of each blot indicate the presence of the proteins and molecular weight markers noted in each panel.

The STAT3 CE epitope interacts with small GTPase and p21Rac1. Autocrine production and signal transduction of IL-6 occurs following persistent Rac1 activity, while small GTPases, including the direct Rac1-STAT3 binding, also regulate STAT3 activation [15,16,17,18,19]. STAT3 binds directly to active (V12 Rac) but not inactive (N17 Rac) Rac1, and this interaction occurs via the effector domain (L61 Rac1) [18]. Since CARP-1 (600–650) interacted with STAT3 [11], we speculated that CARP-1 and p21Rac1 are likely part of a multi-protein complex for activation of the JAK/STAT3 pathway. To test this possibility, we conducted the following studies, utilizing A4 cells that expressed Gst-STAT3 (WT) or Gst-STAT3 (Δ441–480) proteins and HEK293T cells that expressed myc-His-tagged CARP-1 (WT) or CARP-1 (Δ600–650) proteins. For A4 cells, cell lysates were subjected to immunoprecipitation with Gst antibodies. The immunoprecipitants were then analyzed by WB using anti-p21Rac1 antibodies. As shown in Figure 3A, p21Rac1 was present in cells expressing Gst-STAT3 (WT) but not in cells expressing Gst-STAT3 (Δ441–480) proteins. These data suggest that p21Rac1 interacts with the STAT3 CE epitope. We next conducted a similar experiment using HEK293T cells expressing myc-tagged WT CARP-1 or CARP-1 (Δ600–650) proteins. Protein lysates were subjected to immunoprecipitation using anti-myc tag antibodies, followed by WB analyses of immunoprecipitated proteins using anti-p21Rac1 antibodies. This experiment revealed the presence of Rac1 in immunoprecipitants from cells expressing WT, but not mutant (Δ600–650), CARP-1 proteins (Figure 3B). These data suggest that Rac1 interacts with CARP-1 but not with CARP-1 (Δ600–650) mutant protein. These experiments revealed that while p21Rac1 interacted with CARP-1 (WT) or STAT3 (WT) proteins, no interaction of p21Rac1 was noted with the CARP-1 (Δ600–650) or STAT3 (Δ441–480) proteins (Figure 3A,B). Although our co-IP-WB experiments corroborated the interaction between p21Rac1 and STAT3, our findings further revealed that p21Rac1, like CARP-1, interacts with the STAT3 CE epitope. These data suggest that CARP-1 is part of a dynamic complex with STAT3-p21Rac1, which likely regulates STAT3 activation and nuclear shuttling.

**Figure 4:**
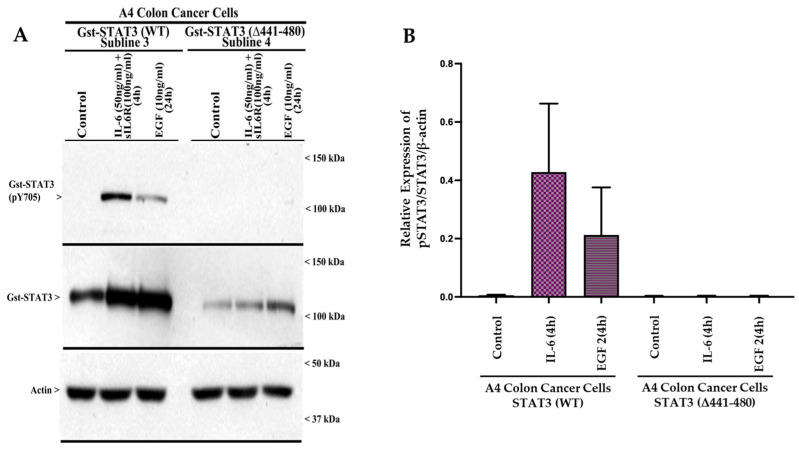
Activation of STAT3 by IL-6 or EGF is abrogated in cells expressing STAT3 (Δ441–480) mutant. (**A**) A4 cells expressing noted Gst-STAT3 plasmids were either treated with DMSO (Control) or treated with the indicated agent at the noted time and dose. The cell lysates were then analyzed by WB for levels of phosphorylated (Y705) and total STAT3 and actin proteins. Arrowheads on the left or right sides of each blot indicate the presence of proteins and molecular weight markers, respectively. (**B**) The WB experiment in (**A**) was repeated (please see Supplementary Figure S2). The histogram represents quantification of the WB signals for pY705-Gst-STAT3. The columns in the bar chart represent the means of two independent experiments: bars, SE.

STAT3 (441–480) regulate STAT3 activation. Since the in-frame deletion of the CE epitope interfered with STAT3 translocation to the nucleus in the absence or presence of IL-6 (Figure 2), we next determined whether and to what extent mutations of the CE epitope amino acids altered/impacted STAT3 activation (Y^705^ phosphorylation) by IL-6 or EGF. In the first instance, we chose A4 STAT3 KO colon cancer cells for the expression of Gst-STAT3 (WT) or Gst-STAT3 (Δ441–480) plasmids. Since these cells lack endogenous STAT3 protein, we expected to determine whether IL-6- or EGF-induced activation of the ectopically expressed mutant STAT3 protein occurs in the absence of any signal from endogenous STAT3. The A4 cells expressing Gst-STAT3 (WT) or Gst-STAT3 (Δ441–480) were either untreated or treated with IL-6 or EGF, as noted in Figure 4. The cell lysates were then analyzed for expression of activated (pY705) STAT3, total STAT3, or Actin proteins. As shown in Figure 4, IL-6 or EGF induced activation of STAT3 in cells expressing Gst-STAT3 (WT) but not in cells that expressed Gst-STAT3 (Δ441–480). These data demonstrate that in-frame deletion of the CE epitope abrogates IL-6- and EGF-induced STAT3 activation.

**Figure 5:**
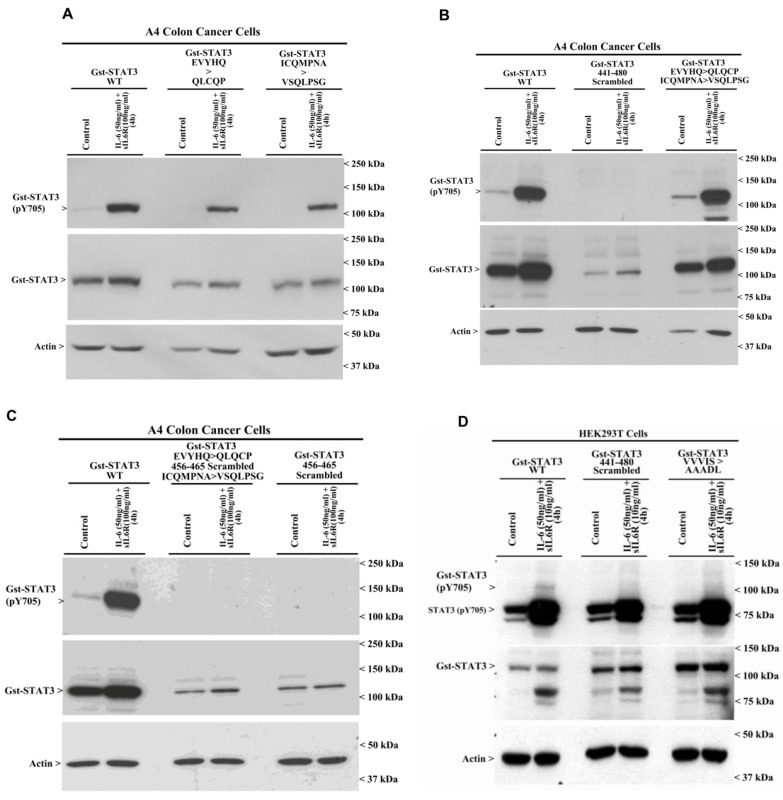

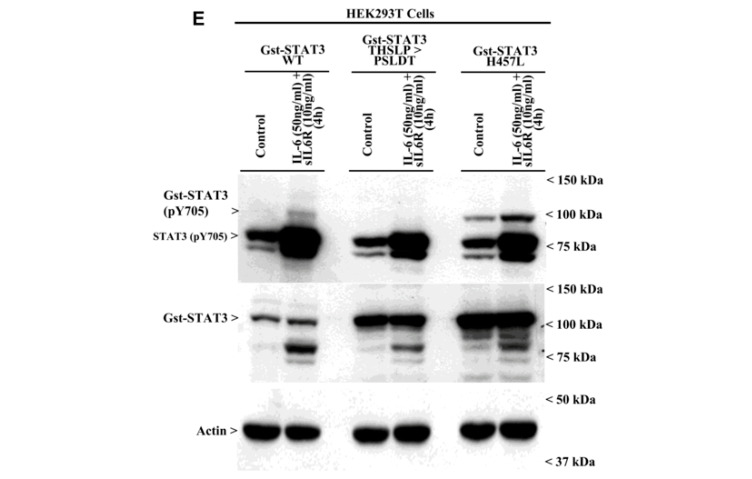
Mapping of the IL-6-responsive minimal epitope of STAT3. A4 colon cancer (**A**–**C**) or HEK293T cells (**D**,**E**) expressing noted Gst-STAT3 plasmids were either treated with DMSO (Control) or treated with the indicated agent at the noted time and dose. The cell lysates were then analyzed by WB for levels of phosphorylated (Y705) and total STAT3 and for actin proteins. Arrowheads on the left or right sides of each blot indicate the presence of proteins and molecular weight markers, respectively.

To further map the sequences within the CE epitope that may regulate STAT3 activation by IL-6, we generated additional Gst-STAT3 constructs where the STAT3 CE epitope was scrambled or harbored different substitution mutations of the amino acids within this CE epitope. Table 1 lists various recombinant plasmids that were utilized for this set of experiments. Notably, the peptide sequences EVYHQ and ICQMPNA within the STAT3 CE epitope were replaced with QLCQP and VSQLPSG, respectively. The peptide sequences QLCQP and VSQLPSG are present in the STAT1 region with homology to the STAT3 CE epitope. These recombinant plasmids were expressed in A4 cells, followed by IL-6 treatment, as described in the Methods. Substitutions of EVYHQ and ICQMPNA within the STAT3 CE epitope to QLCQP and VSQLPSG, respectively, did not abrogate STAT3 activation by IL-6 (Figure 5A,B). Interestingly, scrambling of the entire CE epitope of STAT3 or a decapeptide from positions 456–465 within the CE epitope resulted in loss of STAT3 activation by IL-6 (Figure 5B,C). Although scrambling of STAT3 (441–480) or STAT3 (456–465) abrogated Gst-STAT3 activation in A4 cells, we noted diminished levels of total Gst-STAT3 protein in A4 cells expressing multiple mutant proteins, including Gst-STAT3 (EVYHQ > QLCQP), Gst-STAT3 (ICQMPNA > VSQLPSG), Gst-STAT3 (441–480 Scrambled), Gst-STAT3 (EVYHQ > QLCQP; 456–465 Scrambled; ICQMPNA > VSQLPSG), and Gst-STAT3 (456–465 Scrambled), relative to the levels of Gst-STAT3 (WT) protein (Figure 5A–C). To determine whether IL-6-induced activation of the mutant STAT3 proteins was influenced by the presence of lower levels of respective STAT3 proteins in the A4 cell model, we utilized HEK293T cells in the next set of experiments. HEK293T cells were transiently transfected with plasmids expressing Gst-STAT3 (WT), Gst-STAT3 (441–480 Scrambled), Gst-STAT3 (VVVIS > AAADL), Gst-STAT3 (THSLP > PSLDT), and Gst-STAT3 (H457L). Cells were either untreated or treated with IL-6 followed by WB analysis for the expression of total and activated Gst-STAT3 proteins. As shown in Figure 5D,E, each of the mutants’ plasmids expressed levels of Gst-STAT3 that were relatively higher than those expressed by the Gst-STAT3 (WT) plasmid. Interestingly, although IL-6 stimulated activation of Gst-STAT3 (WT) and Gst-STAT3 (H457L) proteins, it failed to activate Gst-STAT3 (441–480 Scrambled), Gst-STAT3 (VVVIS > AAADL), and Gst-STAT3 (THSLP > PSLDT) proteins (Figure 5D,E). These findings collectively indicate that scrambling of the CE epitope abrogates IL-6-induced STAT3 activation and corroborate our findings shown in Figure 4 above. Further, the pentapeptides THSLP or VVVIS, which are part of the CE epitope within the DNA-binding domain, are likely necessary and sufficient for STAT3 activation by IL-6. A prior report showed that the STAT3 tripeptide VVV (positions 461–463) is also conserved in STAT1 and STAT5, and its substitution to AAA resulted in a loss of DNA binding but not activation of STAT3 by EGF [19]. An additional report has revealed five mutations, including ΔV463 in the STAT3 DNA-binding domain, which was non-functional on its own and showed dominant-negative effects when co-expressed with wild-type STAT3 but did not diminish STAT3 activation [20]. As substitutions of THSLP or VVVIS with PSLDT or AAADL, respectively, result in a loss of IL-6-induced STAT3 activation, our studies indicate that these pentapeptides could be novel small molecules for targeting IL-6-induced STAT3 activation and possibly other activating signals such as EGF.

**Figure 6:**
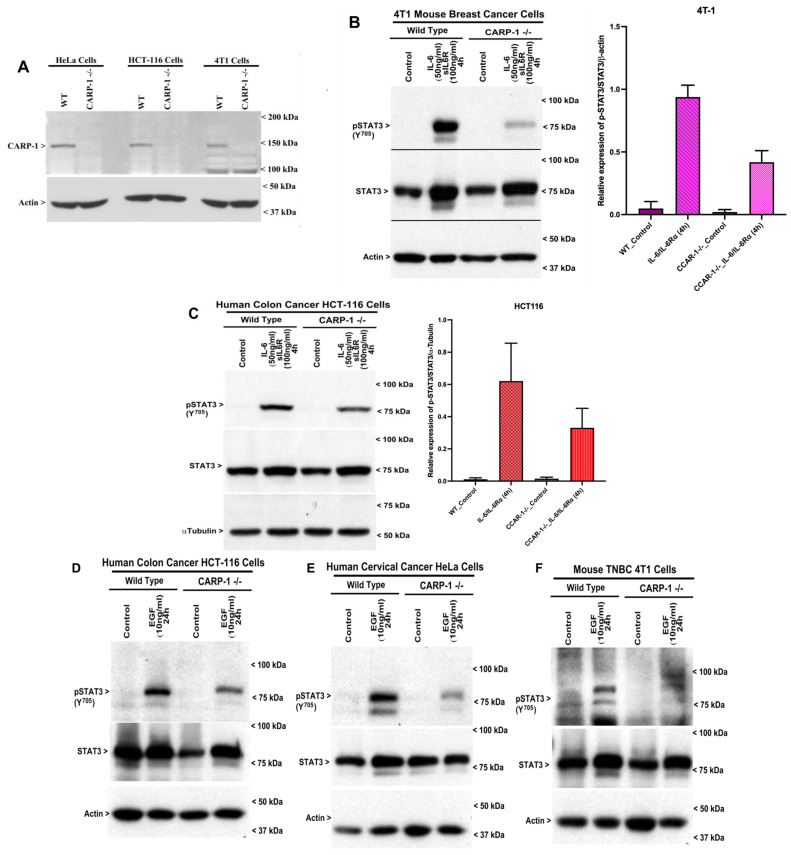
Loss of CARP-1 results in diminished STAT3 activation by IL-6 or EGF. (**A**) Cell lysates from the indicated wild type cells and their respective CARP-1-/- cancer cells were analyzed by WB for CARP-1 and Actin. (**B**–**F**) The indicated wild-type and CARP-1-/- cell lines were either treated with DMSO (Control) or treated with the indicated agent at the noted time and dose. The cell lysates were then analyzed by WB for levels of phosphorylated (Y705) and total STAT3, as well as for actin or α-tubulin proteins. Arrowheads on the left or right side of each blot in panels (**A**–**F**) indicate the presence of the proteins and molecular weight markers, respectively. The WB experiments in (**B**,**C**) were repeated (see Supplementary Figures S3 and S4). The histograms in (**B**,**C**) represent quantification of the WB signals for pY705-Gst-STAT3. The columns in the bar chart represent means of two independent experiments: bars, SE.

CARP-1 loss results in diminished STAT3 activation by IL-6 or EGF. Given that Adriamycin’s presence may not interfere with STAT3 translocation (Figure 2A), deletion of the CE epitope interferes with nuclear translocation of STAT3 and STAT3 activation (Y705 phosphorylation) by IL-6 or EGF (Figure 2 and Figure 4). As STAT3 amino acids 441-480 are part of the CARP-1-interacting CE epitope, we determined whether CARP-1’s presence was necessary for STAT3 activation by IL-6 or EGF. For this purpose, we utilized CARP-1 KO human cervical cancer HeLa cells [10], CARP-1 KO human colon cancer HCT 116, and CARP-1 KO mouse triple-negative breast cancer (TNBC) 4T1 cells, as noted in the Methods. All three CARP-1 KO cell lines, but not their wild-type counterparts, lack CARP-1 (Figure 6A). Next, we either did not treat (Control) or treated each pair of wild-type and CARP-1 KO cells with IL-6 or EGF. As shown in Figure 6B,C, IL-6 treatments provoked robust STAT3 activation in the wild-type 4T1 and HCT-116 cells, while activation of STAT3 was diminished/reduced in their respective CARP-1 KO counterparts that were exposed to IL-6. As also shown in Figure 6D–F, EGF treatments similarly provoked STAT3 activation in the wild-type 4T1, HCT-116, and human cervical cancer HeLa cells. EGF-activated STAT3 levels were diminished in each of the above CARP-1 KO cells relative to the activated STAT3 levels in the EGF-treated respective wild-type cells (Figure 6D–F). Of note is that loss of CARP-1 resulted in a robust decline in STAT3 activation by IL-6 or EGF in cells that have higher EGFR RTK and/or Src activity (HeLa, 4T1), while a somewhat moderate reduction in STAT3 activation by IL-6 or EGF was noted in the CARP-1 KO HCT-116 cells that are known to be driven by Wnt and MAPK pathways. Although CARP-1 also functions as a co-activator of β-catenin in colon cancer cells [6], whether CARP-1 loss would provoke a robust inhibition of STAT3 activation in the colon cancer cells following treatment with the oncogenic Wnt ligands remain to be clarified. Nevertheless, our findings in Figure 6 strongly suggest that CARP-1 regulates STAT3 activation via the oncogenic cytokines EGF or IL-6 that involve CARP-1 interaction with the STATE CE epitope.

**Figure 7:**
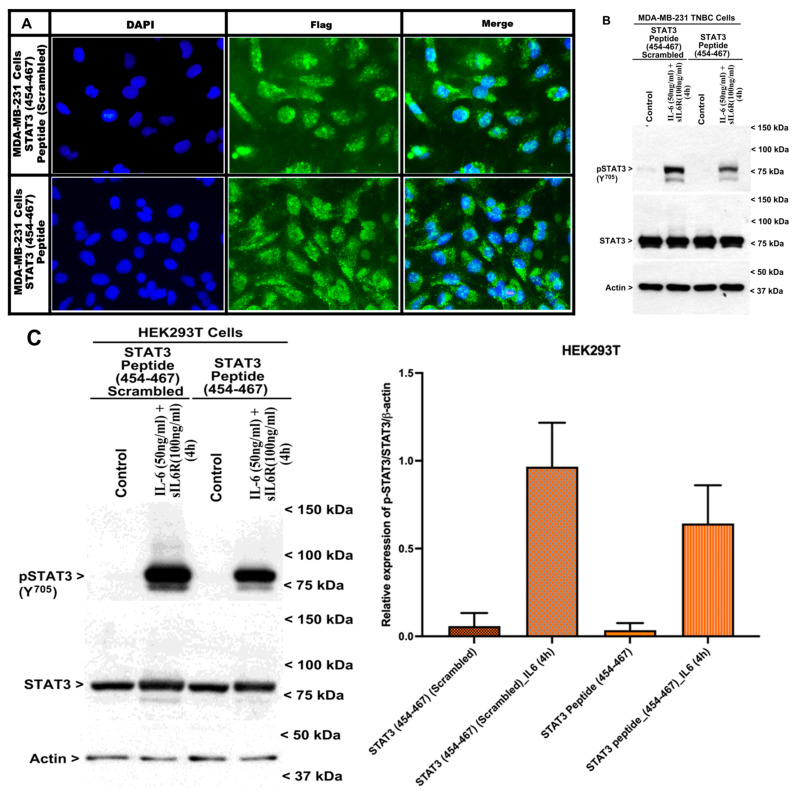

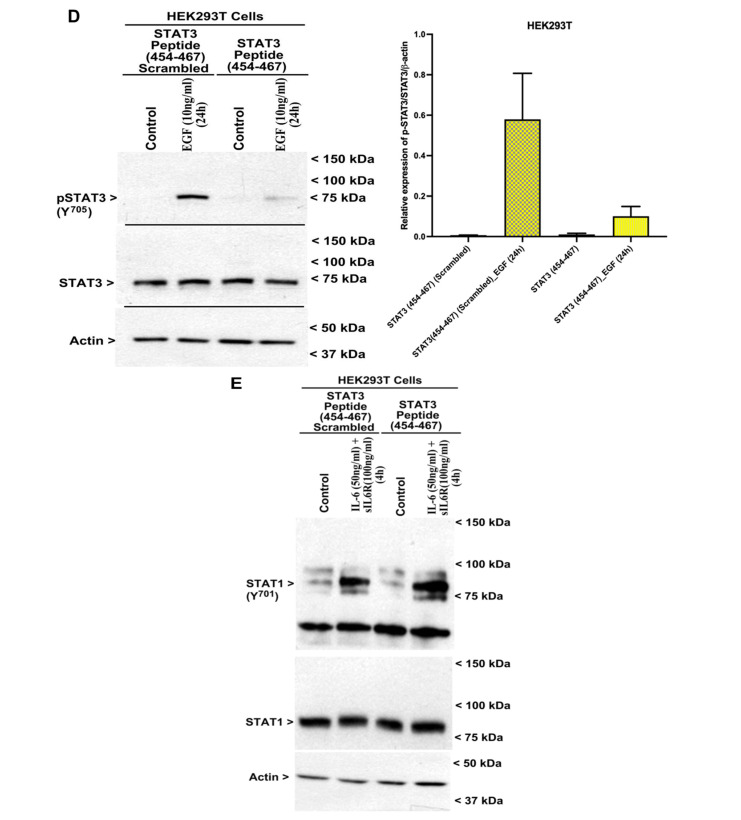
STAT3 (454–467) attenuates IL-6-induced STAT3 activation but not STAT1 activation. (**A**) MDA-MB-231 human TNBC cells were incubated with 500 microgram/mL of each of the TAT-tagged STAT3 (454–467)-Flag peptide or TAT-tagged STAT3 (454–467, scrambled)-Flag peptide for 72 h. Cells were then processed for immunofluorescence staining with a Flag tag antibody (green) and DAPI (blue), as detailed in the Methods. Images were taken using a Zeiss LSM 510 Meta NLO. Magnification: 63×. (**B**–**E**) The indicated cells were first incubated with the noted peptides as in (**A**). The cells were then either treated with DMSO (Control) or with the indicated agent at the noted time and dose. The cell lysates were then analyzed by WB for levels of phosphorylated (Y705) and total STAT3 in panels (**B**–**D**), and for levels of phosphorylated (Y701) and total STAT1 in panel (**E**). Protein loading in (**B**–**E**) was assessed by staining the respective membranes with anti-actin antibodies. Arrowheads on the left or right side of each blot in panels (**B**–**E**) indicate the presence of the proteins and molecular weight markers, respectively. The WB experiments in (**C**,**D**) were repeated (see Supplementary Figures S5 and S6). Histograms in (**C**,**D**) represent quantification of WB signals for pY705-Gst-STAT3. The columns in the bar chart represent the means of two independent experiments: bars, SE.

A STAT3 (454–467) peptide inhibits STAT3 activation by IL-6 or EGF. We next tested whether the incubation of cells with a cell-permeable STAT3 peptide derived from the CE epitope could diminish STAT3 activation by IL-6 or EGF. Multiple studies, including those from our laboratory, have used arginine-rich trans-activator of transcription (TAT) from HIV or other similar protein transduction domain fusion proteins for the import of fusion proteins by cells in vitro and in vivo preclinical models of human disease, including cancer, psoriasis, osteoarthritis, and stroke [21,22,23]. Since expression of Gst-STAT3 (VVVIS > AAADL) or Gst-STAT3 (THSLP > PSLDT) resulted in loss of STAT3 activation by IL-6 in HEK293T cells (Figure 5D,E), we sought to determine whether targeting the region of THSLPVVVIS within the CE epitope of STAT3 would result in a loss of STAT3 activation by IL-6 and/or EGF. For this purpose, we synthesized wild-type and scrambled versions of the STAT3 (454–467) peptide that encompasses the THSLPVVVIS sequence. These peptides were fused to the 6× Histidine and TAT domains at the amino termini and a Flag epitope at the carboxyl termini, as noted in the Methods. First, MDA-MB-231 cells were incubated separately with each peptide, followed by immunofluorescence staining to assess peptide transduction/uptake, as described in the Methods. As shown in Figure 7A, immunofluorescence staining indicated a robust cytosolic presence of the Flag-tagged wild-type and scrambled peptides. Next, since both the wild-type and scrambled peptides robustly transduced MDA-MB-231 cells (Figure 7A) and transfection of the Gst-STAT3 (VVVIS > AAADL) or Gst-STAT3 (THSLP > PSLDT) mutants resulted in loss of STAT3 activation by IL-6 in HEK293T cells (Figure 5D,E), we conducted additional Western blot experiments to determine whether transduction of the wild-type, TAT-tagged THSLPVVVIS peptide also interferes with STAT3 activation by IL-6 or EGF in these cells. Each wild-type or scrambled peptide was pre-incubated with MDA-MB-231 human TNBC or HEK293T cells for 48–72 h. Cells were then untreated or treated for 4 h with IL-6 or EGF. Protein lysates were then analyzed for levels of activated (Y705 phosphorylated) and total STAT3 or activated (Y701) STAT1 and total STAT1 by WB, as noted in the Methods. As shown in Figure 7B,C, the presence of the wild-type peptide provoked a decline in activated STAT3 levels relative to the activated STAT3 levels noted in the cells that were transduced with the scrambled peptide in the IL-6 treated MDA-MB-231 or HEK293T cells. Transduction of the wild-type peptide also provoked a decline in activated STAT3 levels relative to the that noted in the cells that were transduced with the scrambled peptide in the EGF-treated HEK293T cells (Figure 7D). Since several amino acids within the STAT3 CE epitope are conserved in other STAT proteins, particularly STAT1 (Supplementary Figure S7), we tested whether the cell permeable TAT-tagged THSLPVVVIS peptide will also interfere with STAT1 activation by IL-6. The presence of the wild-type TAT-tagged THSLPVVVIS peptide, however, failed to alter STAT1 activation in the IL-6 treated HEK293T cells relative to the activated STAT1 levels noted in the cells that were transduced with the TAT-tagged THSLPVVVIS scrambled peptide (Figure 7E). The data in Figure 7B–D are consistent with our findings shown in Figure 4, Figure 5 and Figure 6 and further highlight the potential of the wild-type STAT3 (454–467) peptide as a novel small-molecule inhibitor of STAT3, but not STAT1, activation by IL-6/EGF. It remains to be clarified whether this peptide functions by competing with STAT3 interaction with CARP-1, p21Rac1, or another endogenous cellular protein(s) and to what extent the cytosolic presence of the TAT-tagged pentapeptide THSLP or VVVIS causes attenuation of STAT3 activation by IL-6 or EGF.

## 4. Discussion

In this report, we elucidate a mechanism of STAT3 activation that involves its interaction with CARP-1/CCAR1. Our prior study revealed that CARP-1 interacted with STAT3, specifically within the CARP-1 (600–650) region, which contains a STAT3-interacting epitope [11]. The interaction between CARP-1 and STAT3 has functional significance, as genotoxic therapy-induced phosphorylation of threonine 627 of CARP-1 attenuated this interaction [11]. Since multiple extrinsic signals such as IL-6 and EGF can activate STAT3, we clarified whether and to what extent STAT3 interaction with CARP-1 also regulated STAT3 activation. To start, we mapped STAT3 sequences that interact with CARP-1. Our mutagenesis studies revealed that STAT3 sequences (441–480), located within the DNA-binding domain, are crucial for its interaction with CARP-1. Treatment with IL-6 or EGF led to significant STAT3 activation, as evidenced by its phosphorylation at tyrosine 705; cells expressing a mutant STAT3 with an in-frame deletion or a scrambled 441–480 region displayed no STAT3 activation in response to IL-6 or EGF. Phosphorylation of STAT3 at tyrosine 705 was shown to promote its dimerization in the cytosol, followed by translocation to the nucleus. Consistent with this, we observed that STAT3 was present in both the cytosol and the nucleus, with increased nuclear presence following IL-6 treatment. Interestingly, the mutant STAT3, which has an in-frame deletion of the 441–480 region, was primarily localized in the cytosol regardless of whether cells were untreated or treated with IL-6.

Multiple members of the Rho family of small GTPases drive activation of STAT proteins in cancer cells, functioning either upstream or downstream depending on the cell type and signaling context [24]. A direct interaction between Rac1 and STAT3 was identified through co-immunoprecipitation with both endogenous and exogenous Rac1, as established through yeast two-hybrid screens (Y2H) [18]. However, this interaction was not observed in all cell types, further emphasizing the context-dependent nature of the Rac1–STAT3 relationship [24]. Our study also uncovered a novel interaction between CARP-1 and the Rho family small GTPase p21Rac1. Initial co-immunoprecipitation experiments indicated a potential interaction of p21Rac1 with CARP-1 (452–654). Since CARP-1 (452–654) contains the STAT3-interacting CARP-1 (600–654) epitope, we found that p21Rac1 did not interact with STAT3 (Δ441–480) or CARP-1 (Δ600–650) mutants. Given that STAT3 (441–480) interacts with CARP-1 (600–650), it suggests that p21Rac1 might be part of a multiprotein complex involved in the activation and translocation of STAT3. Additionally, STAT3 interacts with wild-type CARP-1 and both wild-type and activated (V12) p21Rac1 proteins but does not interact with the dominant negative (N17) mutant of p21Rac1 or the CARP-1 (S^626^, T^627^/AA) mutant [11,18]. Whether wild-type CARP-1 can interact with the dominant-negative (N17) mutant of p21Rac1 remains unclear. Notably, loss of CARP-1 in several cancer cell lines caused loss of STAT3 by IL-6 or EGF, highlighting CARP-1’s critical role in STAT3 signaling.

Whether other Rho family protein(s) support signal-dependent STAT3 activation but need CARP-1 for a robust/optimal STAT3 activation is unclear. Prior reports have indicated a functional link between Rho GTPases and STAT3, including direct interaction of active p21Rac1 with STAT3, as well as indirect STAT3 activation by p21Rac1 through autocrine induction of IL-6; see Refs. [15,16,17,18]. Moreover, our LC-MS/MS analyses [11] revealed the presence of GTPase CDC42 in the proteome bound with CARP-1 (614–638). Further studies will need to clarify whether other GTPases (such as CDC42) also interact with STAT3 CE epitope, to what extent this interaction functions to transduce CARP-1 dependent STAT3 activation, and whether the context(s) of CARP-1/STAT3/CDC42 function overlaps with or is distinct from signaling by oncogenic cytokines IL-6/EGF.

Deletion of STAT3 (441–480) prevents its nuclear localization. Since STAT3 (441–480) is essential for interactions with CARP-1 and p21Rac1, these interactions also facilitate the nuclear import of activated STAT3. While it remains to be clarified whether CARP-1 directly binds to STAT3 and regulates its nuclear translocation, CARP-1 is known to be a peri-nuclear protein [3] with a nuclear localization signal (NLS) at its N-terminus, that is crucial for its nuclear import [25]. Similarly, STAT3 contains an NLS within its coiled-coil domain recognized by the nuclear import machinery, including importin-α3, which facilitates STAT3’s nuclear translocation independent of its activation. Furthermore, active p21Rac1 directly binds with STAT3 and forms a complex with the male germ cell Rac GTPase activating protein (MgcRacGAP). This complex is then targeted to the nuclear pore via the importin α/β pathway for STAT3’s nuclear import [24]. Interestingly, while MgcRacGAP lacks a classical nuclear localization sequence (NLS), mutational analysis identified two basic sequences (182KRR and 199KK) that can function as a bipartite NLS [24]. It is likely that the multiprotein complex of STAT3, p21Rac1, and CARP-1 is involved in the nuclear shuttling of STAT3, depending on the specific cell type and signal context. Therefore, CARP-1 is a critical component of a dynamic multiprotein complex that may facilitate the nuclear import of STAT3, influenced by the cell type and signaling environment.

In various primary cancers and tumor-derived cell lines, STAT1 and STAT3 are constitutively activated [26], and pro-inflammatory cytokines such as IL-6 and EGF are frequently key upstream activators of STAT3. Tyrosine phosphorylation is a prerequisite for STAT protein activation and often promotes their nuclear translocation. The N-terminal domain, the coiled-coil domain, and the DNA-binding domain of STAT proteins function in regulating their nuclear translocation [27]. Moreover, although the CE epitope of STAT3 in its DNA-binding domain is highly conserved with STAT1 (Supplementary Figure S7), it is noteworthy that presence of the STAT3 (454–467) peptide that harbors the THSLPVVVIS sequence results in attenuation of STAT3 but not STAT1 activation by IL-6. Further, our previous LC/MS/MS analyses of proteins interacting with CARP-1 (614–636) showed binding of STAT3 but not STAT1 [11]. Whether and to what extent CARP-1/STAT3/p21Rac1 functions to activate STAT3 but not STAT1 by oncogenic ligands such as IL-6 or EGF remain to be clarified. Our current studies, however, demonstrate that CARP-1 is required for the activation of STAT3 by IL-6 or EGF, in part due to CARP-1’s interaction with a novel epitope within the STAT3 DNA-binding domain. This interaction leads to the phosphorylation of STAT3 at tyrosine 705 (Y705), as well as its nuclear translocation, suggesting that CARP-1 acts as a transducer of pro-inflammatory signaling via STAT3.

Further fine mapping of the CARP-1 and p21Rac1-interacting epitope of STAT3 revealed that a shorter, ten-amino acid peptide from positions 456–465 may be both sufficient and necessary for STAT3 activation by IL-6 or EGF. IL-6 failed to activate STAT3 in cells expressing a mutant STAT3 protein with a scrambled 456–465 peptide (Figure 5C). This peptide contains the DNA-binding tripeptide VVV, which is highly conserved between STAT3 and STAT1 [19]. While substituting the tripeptide VVV with AAA abolished EGF-induced DNA binding of STAT3, it did not affect Y705 phosphorylation [19]. Interestingly, substituting the pentapeptide VVVIS (461–465) to AAADL abolished STAT3 Y705 phosphorylation by IL-6 (Figure 5D), likely preventing its translocation to the nucleus and subsequent DNA binding. Moreover, the 456–465 peptide of STAT3 also harbors the pentapeptide THSLP (456–460). When this THSLP pentapeptide was substituted with PSLDT, it also resulted in the loss of STAT3 Y705 phosphorylation by IL-6. Thus, scrambling STAT3 441–480 or 456–465, as well as substitutions within the sequences of STAT3 456–460 or 461–465, result in loss of STAT3 Y705 phosphorylation by IL-6 (Figure 5E). However, substitutions of sequences EVYHQ (444–448) and ICQMPNA (467–473) for QLCQP and VSQOPSG, respectively, did not abrogate STAT3 Y705 phosphorylation by IL-6 (Figure 5A,B). This suggests that the STAT3 456–465, 456–460, and 461–465 peptide regions within the CARP-1-interacting epitope likely have a unique mechanism of STAT3 activation by IL-6. Given that the CARP-1-interacting epitope of STAT3 also interacts with small GTPase p21Rac1, it remains to be clarified whether scrambling or substitutions disrupt STAT3’s interactions with CARP-1 and/or p21Rac1. These findings indicate that the sequences 456–465, 456–460, and 461–465 of STAT3 could potentially be novel targets for modulating STAT3 activation (Y705 phosphorylation) by IL-6 and possibly by other oncogenic molecules like EGF. For proof of concept, we tested this hypothesis as follows. We treated mammalian cells with a synthetic STAT3 peptide (454–467) that also contained an arginine-rich TAT epitope at its amino terminus to facilitate transport across the plasma membrane. Although TAT-STAT3 (454–467) or its scrambled version localized to the cytosol, only TAT-STAT3 (454–467) and not its scrambled version reduced STAT3 Y705 phosphorylation without affecting STAT1 Y701 phosphorylation in cells treated with IL-6. These preliminary findings suggest that STAT3 (456–465) participates in a dynamic mechanism(s) that likely involves its interaction(s) with cytoplasmic factors(s) for effective signal transduction by cytokines such as IL-6 or EGF. Ectopic supplementation of TAT-STAT3 (454–467) that results in its cytoplasmic accumulation likely also results in targeted disruption of STAT3 interactions with cytoplasmic protein(s) that may be necessary for STAT3 activation during IL-6 signaling. Further studies are needed to determine whether IL-6-induced activation of STAT3 involves interaction between the 456–465 region and CARP-1, p21Rac1, or other yet-to-be-identified factors(s) are crucial for effective oncogenic signal transduction by STAT3.

## Figures and Tables

**Table 1:** List of various Gst-STAT3 plasmids that were transfected into A4 colon cancer or HEK293T cells. ND, Not Done.

Reconstitution Experiments			
A4 STAT3 -/- Cells	IL-6 Inducibility (Phospho-Y705)	Src Inducibility (Phospho-Y705)	Nuclear Translocation
Gst-STAT3 (WT)	Yes	Yes	Yes
Gst-STAT3 (Δ441–480)	No	No	No
Gst-STAT3 (Δ441–465)	No	No	No
Gst-STAT3 (Δ456–480)	No	No	No
Gst-STAT3 (441–480 Scrambled)	No	ND	ND
Gst-STAT3 (456–465 Scrambled)	No	ND	ND
Gst-STAT3 (444 EVYHQ 448 > QLCQP)	Yes	ND	ND
Gst-STAT3 (467 ICQMPNA 473 > VSQLPSG)	Yes	ND	ND
Gst-STAT3 (EVYHQ > QLCQP; 456–465 Scrambled; ICQMPNA > VSQLPSG)	No	ND	ND
**HEK293T Cells**	**IL-6 Inducibility (Phospho-Y705)**	**Src Inducibility (Phospho-Y705)**	**Nuclear Translocation**
Gst-STAT3 (WT)	Yes	ND	ND
Gst-STAT3 (Δ441–480)	No	ND	ND
Gst-STAT3 (Δ441–465)	No	ND	ND
Gst-STAT3 (Δ456–480)	No	ND	ND
Gst-STAT3 (441–480 Scrambled)	No	ND	ND
Gst-STAT3 (456–465 Scrambled)	No	ND	ND
Gst-STAT3 (444 EVYHQ 448 > QLCQP)	Yes	ND	ND
Gst-STAT3 (467 ICQMPNA 473 > VSQLPSG)	Yes	ND	ND
Gst-STAT3 (EVYHQ > QLCQP; ICQMPNA > VSQLPSG)	Yes	ND	ND
Gst-STAT3 (EVYHQ > QLCQP; 456–465 Scrambled; ICQMPNA > VSQLPSG)	No	ND	ND
Gst-STAT3 (456 THSLP 460 > PSLDT)	No	ND	ND
Gst-STAT3 (461 VVVIS 465 > AAADL)	No	ND	ND
Gst-STAT3 (H457/L)	Yes	ND	

## Data Availability

The original contributions presented in this study are included in the article/Supplementary Material. Further inquiries can be directed at the corresponding author.

## Supplementary Materials

The following supporting information can be downloaded at: https://www.mdpi.com/article/10.3390/cells15090755/s1, Figure S1: Generation of Gst-STAT3 sublines; Figure S2: Activation of STAT3 by IL-6 or/ EGF is abrogated in cells expressing STAT3 (Δ441–480) mutants; Figure S3: (In support of Figure 6B) Loss of CARP-1 results in diminished STAT3 activation by IL-6; Figure S4: (In support of Figure 6C) Loss of CARP-1 results in diminished STAT3 activation by IL-6; Figure S5: (In support of Figure 7C) STAT3 (454–467) attenuates IL-6-induced STAT3 activation; Figure S6: (In support of Figure 7D) STAT3 (454–467) attenuates IL-6-induced STAT3 activation; Figure S7: Alignments of CARP-1-binding STAT3 Peptide with Human STAT Proteins.
