# Supplementary material for: A Novel Mechanism of STAT3 Activation by Oncogenic Signaling
Source: Cells. 2026 Apr 23;15(9):755. doi: 10.3390/cells15090755 (PMC13162942; doi:10.3390/cells15090755)
Supplement: Supplementary file 1 [file cells-15-00755-s001.zip › cells-3981546-supplementary.pdf]

A

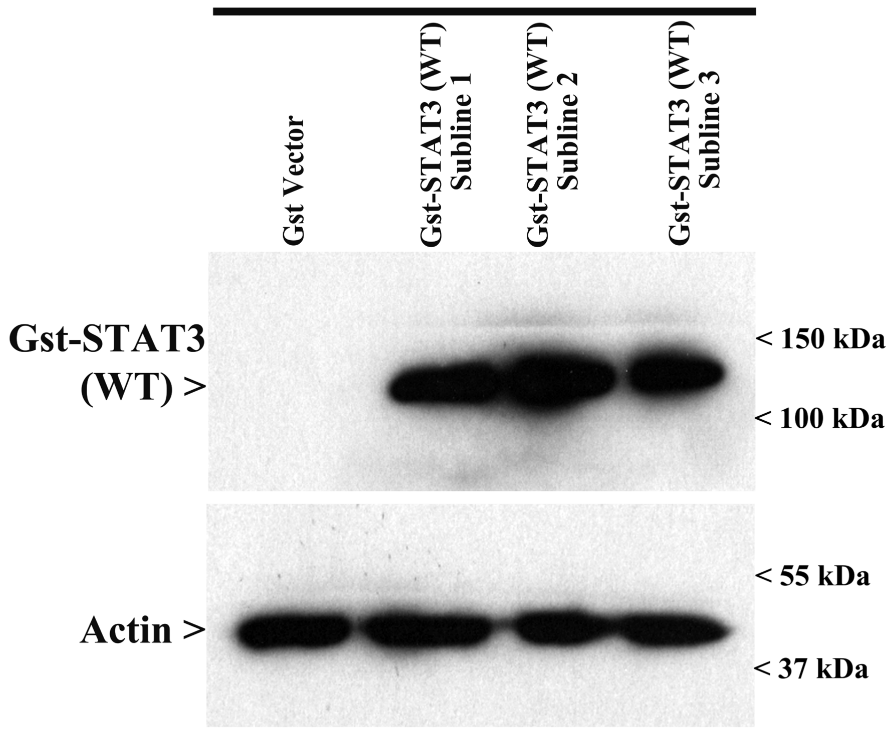

B

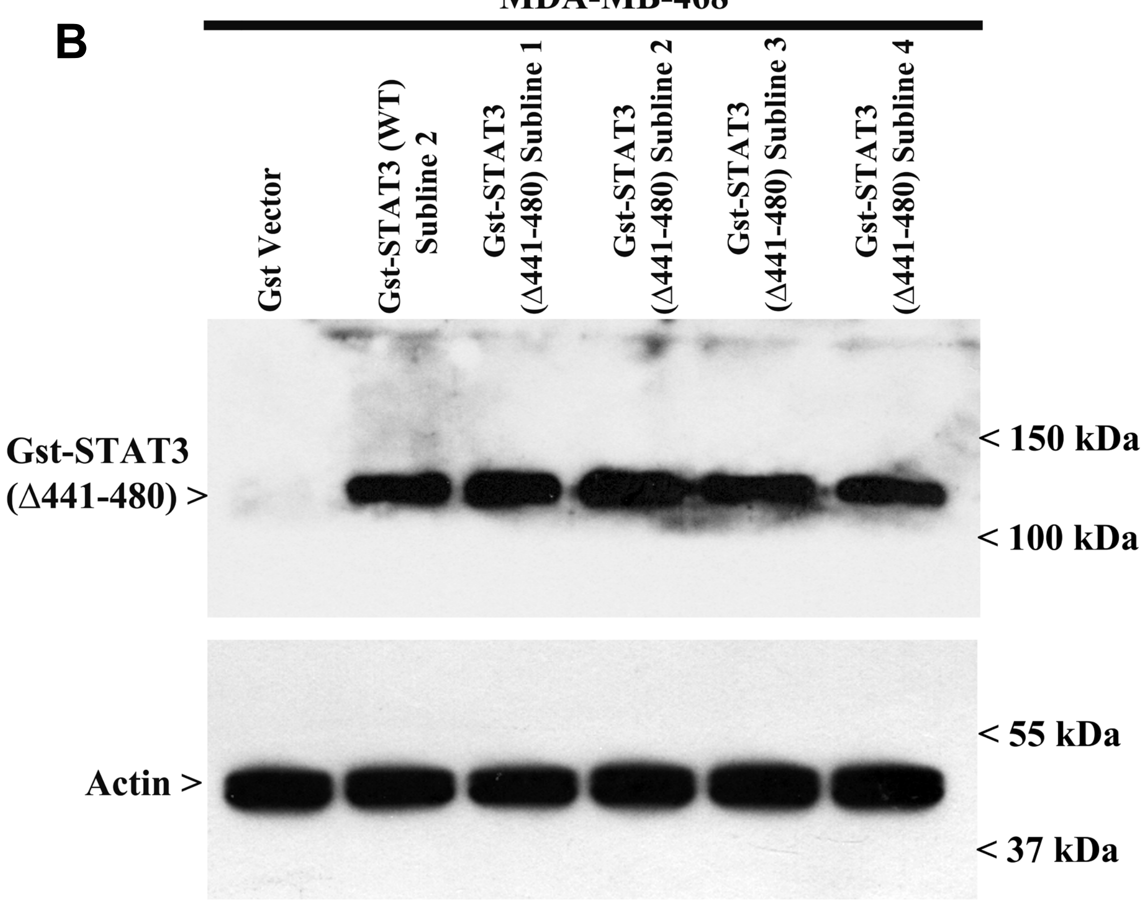

Supplementary Figure S1

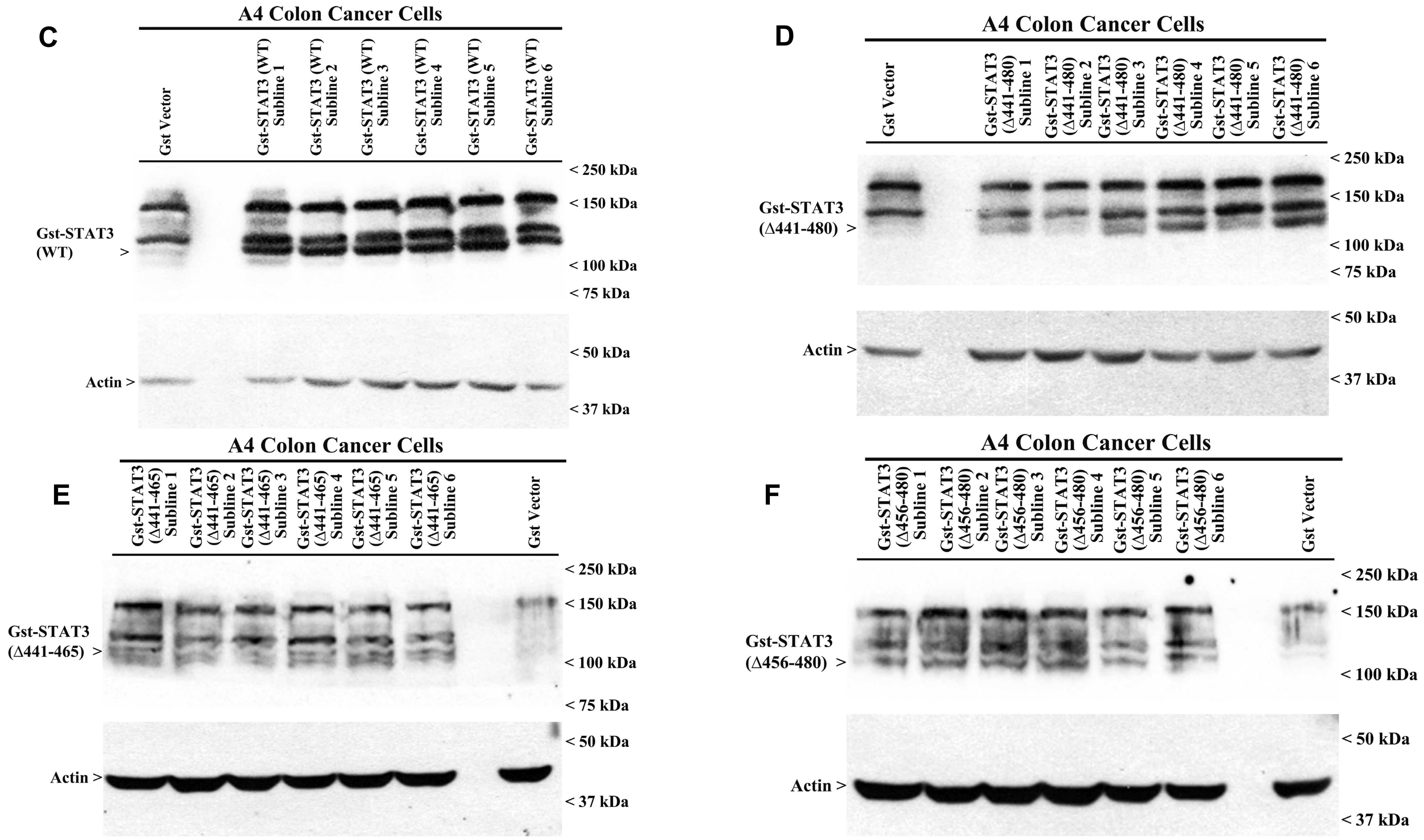

C
A4 Colon Cancer Cells
Gst Vector
Gst-STAT3 (WT) Subline 1
Gst-STAT3 (WT) Subline 2
Gst-STAT3 (WT) Subline 3
Gst-STAT3 (WT) Subline 4
Gst-STAT3 (WT) Subline 5
Gst-STAT3 (WT) Subline 6
Gst-STAT3 (WT) >
Actin >
< 250 kDa
< 150 kDa
< 100 kDa
< 75 kDa
< 50 kDa
< 37 kDa
D
A4 Colon Cancer Cells
Gst Vector
Gst-STAT3 (Δ441-480) Subline 1
Gst-STAT3 (Δ441-480) Subline 2
Gst-STAT3 (Δ441-480) Subline 3
Gst-STAT3 (Δ441-480) Subline 4
Gst-STAT3 (Δ441-480) Subline 5
Gst-STAT3 (Δ441-480) Subline 6
Gst-STAT3 (Δ441-480) >
Actin >
< 250 kDa
< 150 kDa
< 100 kDa
< 75 kDa
< 50 kDa
< 37 kDa
E
A4 Colon Cancer Cells
Gst-STAT3 (Δ441-465) Subline 1
Gst-STAT3 (Δ441-465) Subline 2
Gst-STAT3 (Δ441-465) Subline 3
Gst-STAT3 (Δ441-465) Subline 4
Gst-STAT3 (Δ441-465) Subline 5
Gst-STAT3 (Δ441-465) Subline 6
Gst Vector
Gst-STAT3 (Δ441-465) >
Actin >
< 250 kDa
< 150 kDa
< 100 kDa
< 75 kDa
< 50 kDa
< 37 kDa
F
A4 Colon Cancer Cells
Gst-STAT3 (Δ456-480) Subline 1
Gst-STAT3 (Δ456-480) Subline 2
Gst-STAT3 (Δ456-480) Subline 3
Gst-STAT3 (Δ456-480) Subline 4
Gst-STAT3 (Δ456-480) Subline 5
Gst-STAT3 (Δ456-480) Subline 6
Gst Vector
Gst-STAT3 (Δ456-480) >
Actin >
< 250 kDa
< 150 kDa
< 100 kDa
< 50 kDa
< 37 kDa

Supplementary Figure S1: Generation of Gst-STAT3 sublines. A-F. Cell lysates from the MDA-MB-468 human TNBC (A, B) and A4 colon cancer cells (C-F) expressing indicated plasmids were analyzed by WB for STAT3 and Actin proteins. Arrowheads on the left or right side, respectively, of each blot in panels A-F indicate presence of the proteins and molecular weight markers.

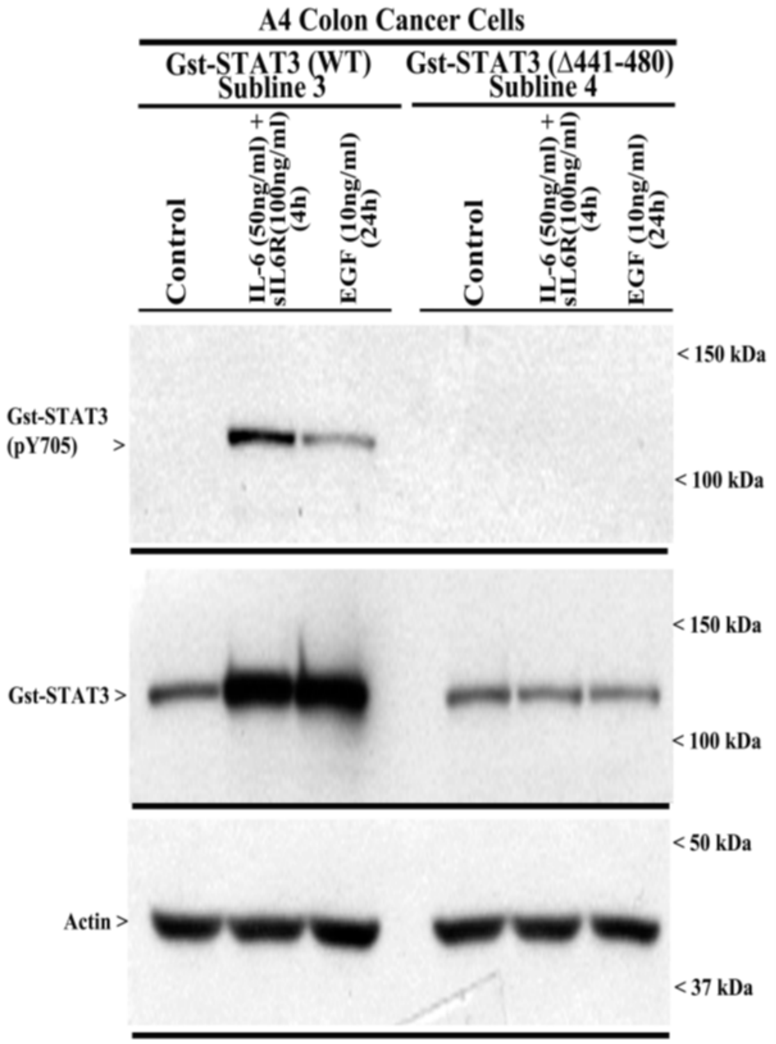

Supplementary Figure S2. (In support of figure 4). Activation of STAT3 by IL-6 or/ EGF is abrogated in cells expressing STAT3 (Δ441-480) mutants. A4 cells expressing noted Gst-STAT3 plasmids were either treated with DMSO (Control), treated with noted time and dose of the indicated agent. The cell lysates were then analyzed by WB for levels of phosphorylated (Y705) and total STAT3, and actin proteins. Arrowheads on the left or right sides of each blot indicate the presence of proteins and molecular weight markers.

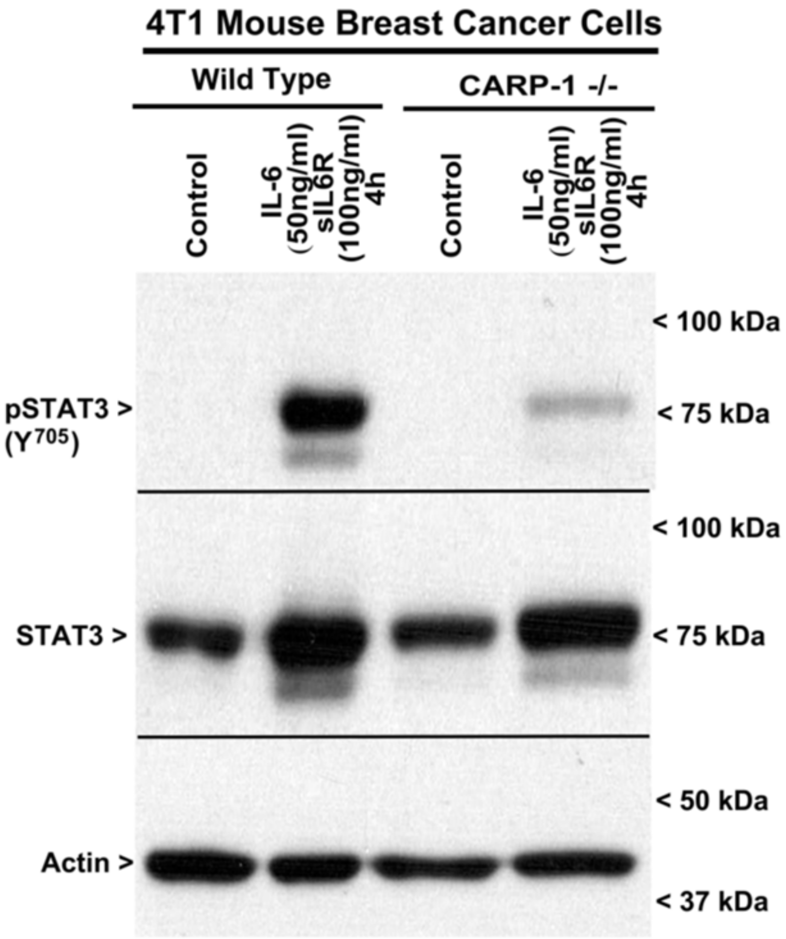

Supplementary Figure S3. (In support of figure 6B). Loss of CARP-1 results in diminished STAT3 activation by IL-6. Indicated wild-type and CARP-1-/- cell lines were either treated with DMSO (Control) or treated with the noted time and dose of the indicated agent. The cell lysates were then analyzed by WB for levels of phosphorylated (Y705) and total STAT3, and for actin proteins. Arrowheads on the left or right side, respectively, of each blot indicate the presence of the proteins and molecular weight markers.

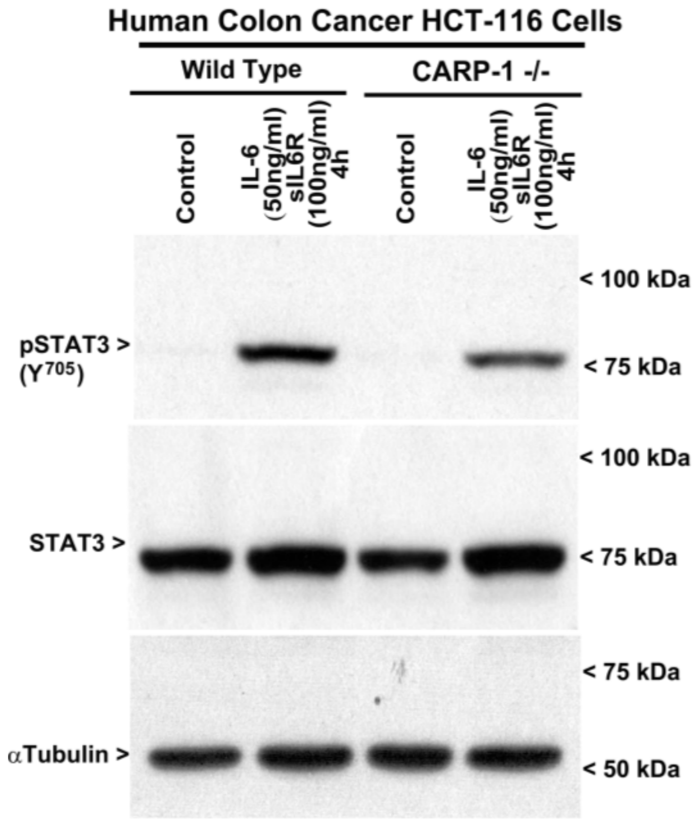

Supplementary Figure S4. (In support of figure 6C). Loss of CARP-1 results in diminished STAT3 activation by IL-6. Indicated wild-type and CARP-1-/- cell lines were either treated with DMSO (Control) or treated with the noted time and dose of the indicated agent. The cell lysates were then analyzed by WB for levels of phosphorylated (Y705) and total STAT3, and for αtubulin proteins. Arrowheads on the left or right side, respectively, of each blot indicate the presence of the proteins and molecular weight markers.

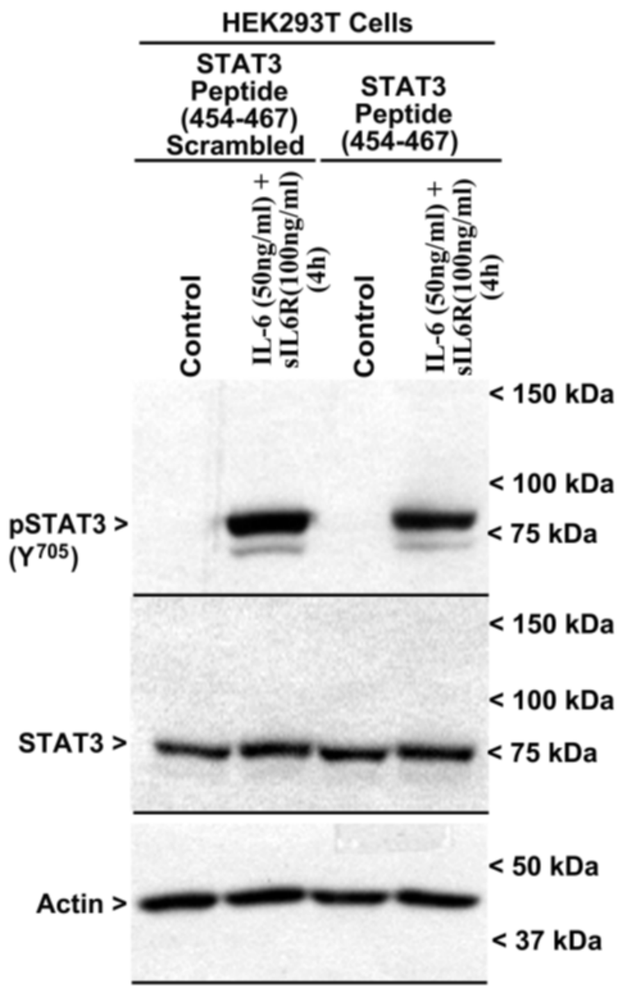

Supplementary Figure S5. (In support of Figure 7C). STAT3 (454-467) attenuates IL-6-induced STAT3 activation. Indicated cells were first incubated with noted peptides and then either treated with DMSO (Control) or with the indicated agent at the noted time and dose. The cell lysates were then analyzed by WB for levels of phosphorylated (Y705) and total STAT3. Protein loading was assessed by staining the respective membranes with anti-actin antibodies. Arrow-heads on the left or right side, respectively, of each blot indicate the presence of the proteins and molecular weight markers.

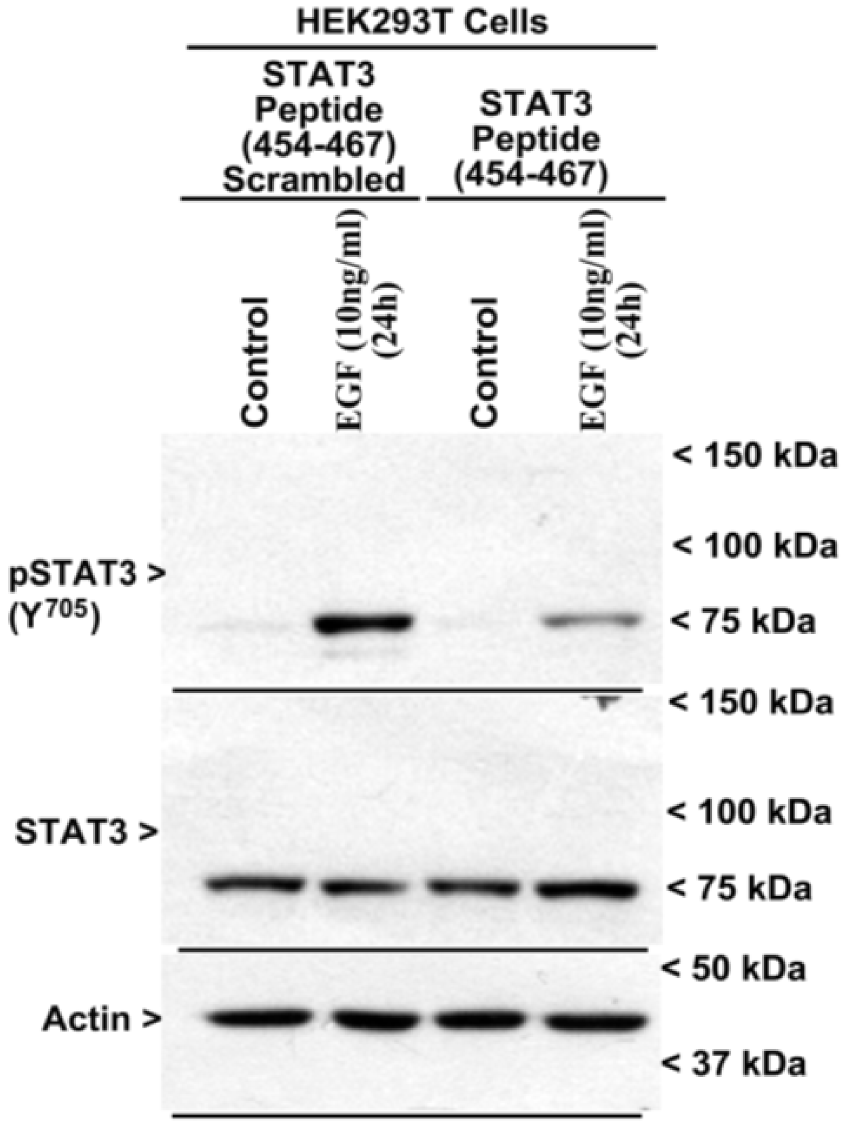

Supplementary Figure S6. (In support of Figure 7D). STAT3 (454-467) attenuates IL-6-induced STAT3 activation. Indicated cells were first incubated with noted peptides and then either treated with DMSO (Control) or with the indicated agent at the noted time and dose. The cell lysates were then analyzed by WB for levels of phosphorylated (Y705) and total STAT3. Protein loading was assessed by staining the respective membranes with anti-actin antibodies. Arrow-heads on the left or right side, respectively, of each blot indicate the presence of the proteins and molecular weight markers.

hSTAT3 CE Epitope	FETEVYHQGLKIDLETHSLPVVVIS N I C QMPNA WAS I LW
hSTAT1 alpha/beta	FETQLCQPGLVIDLETT SLPVVVIS N VS Q L PS G WAS I LW
hSTAT2	FTVKYTYQGLKQELKTDTLPVVI IS NMNQ L S I A WAS VLW
hSTAT5A	LVFQVKTLSLPVVVIV H GS Q DHNA T AT VLW
hSTAT5B	LVFQVKTLSLPVVVIV H GS Q DNNA T AT VLW
hSTAT6	LPI QLQALSLPLVVIV H GN Q DNNA K AT I LW

Supplementary Figure S7. Alignments of CARP-1-binding STAT3 Peptide with Human STAT Proteins. Amino acid conservation among the sequences within the STAT3 (454-467) peptide are highlighted in color.
